# Correlation between Inter-Blink Interval and Episodic Encoding during Movie Watching

**DOI:** 10.1371/journal.pone.0141242

**Published:** 2015-11-03

**Authors:** Young Seok Shin, Won-du Chang, Jinsick Park, Chang-Hwan Im, Sang In Lee, In Young Kim, Dong Pyo Jang

**Affiliations:** 1 Department of Biomedical Engineering, Hanyang University, Seoul, Korea; 2 Department of Theater and Film, Hanyang University, Seoul, Korea; University of Waterloo, CANADA

## Abstract

Human eye blinking is cognitively suppressed to minimize loss of visual information for important real-world events. Despite the relationship between eye blinking and cognitive state, the effect of eye blinks on cognition in real-world environments has received limited research attention. In this study, we focused on the temporal pattern of inter-eye blink interval (IEBI) during movie watching and investigated its relationship with episodic memory. As a control condition, 24 healthy subjects watched a nature documentary that lacked a specific story line while electroencephalography was performed. Immediately after viewing the movie, the subjects were asked to report its most memorable scene. Four weeks later, subjects were asked to score 32 randomly selected scenes from the movie, based on how much they were able to remember and describe. The results showed that the average IEBI was significantly longer during the movie than in the control condition. In addition, the significant increase in IEBI when watching a movie coincided with the most memorable scenes of the movie. The results suggested that the interesting episodic narrative of the movie attracted the subjects’ visual attention relative to the documentary clip that did not have a story line. In the episodic memory test executed four weeks later, memory performance was significantly positively correlated with IEBI (p<0.001). In summary, IEBI may be a reliable bio-marker of the degree of concentration on naturalistic content that requires visual attention, such as a movie.

## Introduction

Investigation of eyelid activity has indicated that eye blinks can be distinguished into three types in humans: spontaneous, reflexive, and voluntary [1]. Of these, spontaneous eye blinks can reflect cognitive state or emotions in humans [2]. Under the conditions of everyday life, a human blinks approximately 15 times per minute [3]. Eye blinks can interrupt the acquisition of visual information for approximately 400 ms per blink [4]. Thus, human eye blinking is cognitively suppressed to minimize the loss of visual information for important real-world events [5–9].

Despite the relationship between eye blinks and cognitive state, the effect of eye blinks on cognition in real-world environments has received limited research attention [10]. The majority of previous work has focused on the specific timing of eye blinks in response to stimuli or on tasks performed in highly controlled experimental settings. Hall et al. has reported that eye blinks are more likely to occur at certain points in time, such as when a punctuation mark is reached when reading text [11]. Similarly, blinks tend to occur immediately before and after the presentation of stimuli during a task [12–14]. In addition, spontaneous blinks are highly synchronized at the end of a performance, during the absence of a character, and during the repetition of a scene that may require less attention [15]. However, it is unlikely that clear instances of stimulus presentation will be present in the varying flow of visual attention in daily life. It is also difficult to interpret eye blinks as an attentional component under natural conditions because blinking can be performed in response to environmental stimuli such as dryness, as a reflex, or voluntarily. Furthermore, to understand blink suppression as it relates to visual attention, it is important to consider when eye blinks are suppressed rather than when eye blinks occur during naturalistic conditions. Therefore, we hypothesized that inter-eye blink interval (IEBI) is a more reliable biomarker of the relationship between blink suppression and cognitive functions such as memory.

In this study, we investigated the temporal pattern of IEBI when subjects watched a movie that provided naturalistic cognitive information [16–18]. In addition, the relationship between episodic memory and IEBI was analyzed to evaluate visual attention and determine the effects of eye blink suppression on episodic memory.

## Materials and Methods

### Ethics statement

All of the subjects provided written informed consent and were given a small financial incentive for participating in the study, which was approved by the Institutional Review Board of Hanyang University.

### Subjects

Twenty-four healthy subjects (11 males and 13 females) were recruited for this experiment. The subjects ranged from 20 to 29 years of age. All subjects reported normal or corrected-to-normal visual acuity using glasses, as well as normal auditory function. None of the subjects had any history of major eye problems. We excluded the subjects using contact lenses to prevent fatigue or dryness caused by contact lenses. The humidity in the experiment environments was kept at approximately 50% to minimize that drying of the cornea as this could influence the rate of blinking. Subjects were unaware as to the purpose of the experiment until after the experiment.

### Experimental design

Subjects were instructed to perform three tasks during the experiment. The first task was to watch a 2-hour movie, the Korean movie ‘The Chaser’ (MOVIE) [19]. Subjects who had seen the movie before the experiment were excluded. The second task was to watch a 5-minute landscape video from a nature documentary in which beautiful scenes were displayed without any story line (CONTROL). The final task instructed participants to look at a black screen without any visual stimuli for 5 minutes (REST). Also, they took a 5-minute rest between tasks, and the tasks were randomly ordered. The participants were monitored by a camera to check for fatigue during each condition. In the monitoring, no one fell asleep during the experiment.

All tasks were performed using a wide screen in conjunction with a beam projector (LG PW700) in a darkroom. The projector had a HD resolution of 1280 × 800 and a 100,000:1 contrast. The 1600 mm (W) 1200 mm (H) wide screen was located 1.0m in front of the subject. The stimuli subtended 80 degrees of the subject’s field of view. The height of the seat was adjusted so that the screen and speaker were at the same height as the subject’s eyes and ears. Sounds were presented via a Britz Bluetooth speaker system (BZ-D20 Reflex,) with 5W output power. The distance from the speaker to the subject was 1.1 m. It was behind of center of screen. Speaker volume was adjusted to the comfort level of each subject before the experiment began, because extremely loud sounds were likely to cause a reflex eyeblink.

Immediately after the three tasks, the subjects were asked to report the most memorable scene during film watching in order to find the relationship between memorable scenes and temporal pattern of IEBIs and to understand common interesting scenes across subjects. They were free to select any one scene in the movie which they felt to be the most memorable and they were asked to describe the scene.

An episodic memory test was performed four weeks after the experimental tasks took place. The episodic memory test was composed of 32 items. Each item included a still shot and two questions. Still shots representing a scene were selected to serve as visual cues for recall. Thirty two scenes from the movie were randomly selected out of 99 potential scenes. A scene was defined as a section of the move taking place in one location over a continuous period of time (e.g. a love scene, an action scene, a car chase scene). Each scene was assigned a number and 32 were selected randomly. Subjects were given two questions to answer with regard to each scene. Q1: recall and describe at which point of the movie the given shot is shown. And Q2: score each scene according to how memorable they are with a scale of 1 (not at all) to 5 (perfectly).

### Data acquisition and analysis

**Fig 1:**
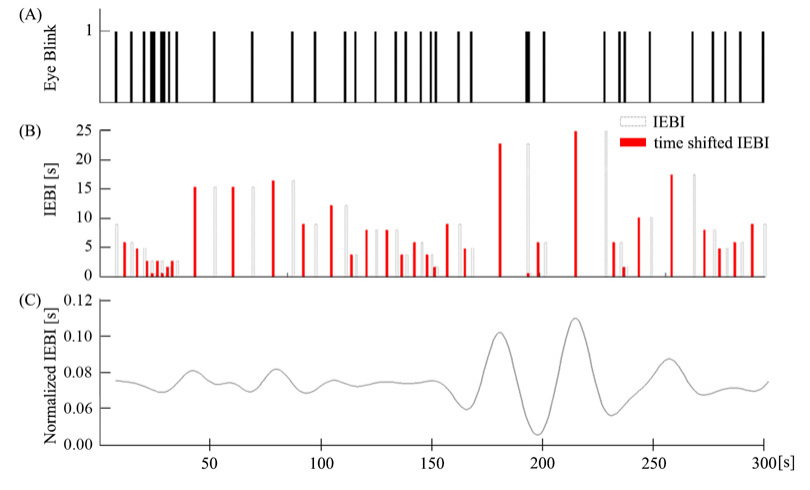
Pre-processing of eye blink data. (A) Raster plot of eye blinks. (B) IEBI plot aligned to the center time point (red bar) as a representative sample of IEBIs. (C) Continuous plot of IEBI filtered with a Gaussian kernel after normalization to the average IEBI.

Electroencephalography (EEG) signals were recorded over 12 channels using g.MOBIlab (g.tec Medical Engineering GmbH, Graz, Austria). Eye blink data were measured from electromyography signals in EEG channels. The eye blink component was extracted from the FP1 and FP2 electrodes by a template-matching algorithm that used dynamic positional warping to identify specific patterns in the EEG [20]. The data were analyzed offline using MATLAB (The MathWorks, Inc., Natick, MA, USA). After detecting eye blinks, we calculated the number of eye blinks per second in order to reduce data size. The number of eye blinks per second was converted to IEBI and aligned to the center time point in order to depict a representative sampling of IEBIs (Fig 1). To compare the three tasks (REST, CONTROL, and MOVIE), we employed the Kolmogorov-Smirnov test using the kstest2() Matlab function which does not assume a normal distribution. For further analysis, each subject’s MOVIE IEBI was normalized to the average of their MOVIE IEBI. Additionally, IEBI data were filtered with a Gaussian kernel. We made a 60 point Gaussian window with 4 proportional to the reciprocal of the standard deviation using the gausswin() Matlab function. The filtered IEBIs of all subjects were averaged. A random permutation test [21] was used to establish statistical significance (p < 0.01) of the peak time series. In addition, Pearson’s correlation was used to validate the relationship between IEBI and memory capacity for 32 randomly selected scenes from the movie.

## Results

**Fig 2:**
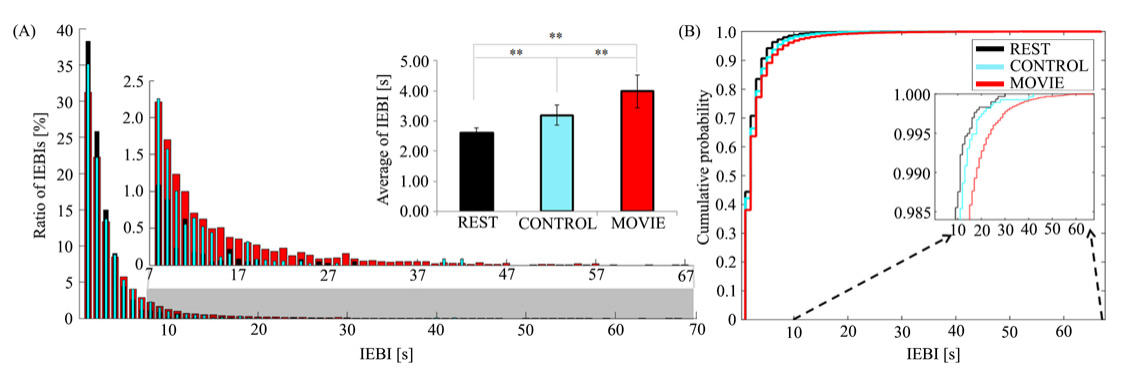
Statistical comparisons and cumulative distribution in three conditions. (A) The graph shows the average of IEBIs and histogram of IEBIs for each of the three conditions (Fig 2 and S1 Dataset). The portion of the histogram between 8 and 67 s has been magnified to more clearly illustrate the distribution of long IEBIs. (B) Cumulative distribution functions for each of the three conditions. The red, sky-blue and black lines show MOVIE, CONTROL and REST conditions, respectively. (*p* < 0.01** Denotes a statistically significant difference.)

The mean IEBIs in REST and CONTROL conditions were 2.6 ± 0.8(SD) s and 3.2 ± 1.6 (SD) s respectively, increasing to 4.0 ± 2.6 (SD) s during the movie watching. The changes of IEBIs across conditions were more clearly visible in the distribution of IEBIs, as shown in Fig 2A. IEBI were more widely distributed within the MOVIE condition compared with the CONTROL and REST conditions. As mentioned above, Kolmogorov-Smirnov tests were employed to compare the statistical difference across conditions. Statistically significant differences were revealed between MOVIE and CONTROL (p < 0.01, k = 0.0408), MOVIE and REST (p < 0.01, k = 0.0708) and CONTROL and REST (p < 0.01, k = 0.0434). And the medians in MOVIE, CONTROL and REST were 2. Interquartile ranges of these conditions also were 2.

Next we considered cumulative distribution function plots (CDF) to assess the shift in distribution across conditions. The difference in distribution between specific IEBIs with duration were clearly found as shown in Fig 2B. For further analysis, we divided the data into “short” or “long” IEBIs [22]. When the subjects were watching a movie, the number of long IEBIs (> 8 sec) increased, while the number of short IEBIs (< 8 sec) decreased. Also, long IEBIs exceeding 40 seconds found in MOVIE conditions only as shown in Fig 2. In particular, very long IEBIs with durations longer than one minute were present in the MOVIE condition. Similarly, the number of short IEBIs (< 8 sec) was significantly reduced in the MOVIE condition (mean: 91.8, SD: 11.8) compared to the CONTROL (mean: 95.7, SD: 6.7, t = -2.310, df = 23, p<0.05, 2-tailed) and REST (mean: 98.1, SD: 2.9, t = -2.442, df = 23, p<0.05, 2-tailed) conditions. In contrast, the number of long IEBIs significantly increased in the MOVIE condition (mean: 8.1, SD: 11.8) compared to the CONTROL (mean: 4.3, SD: 6.7, t = 2.310, df = 23, p<0.05, 2-tailed) and REST (mean: 1.9, SD: 2.9, t = 2.442, df = 23, p<0.05, 2-tailed) conditions.

### Relationship between IEBI and memorable movie scenes

**Fig 3:**
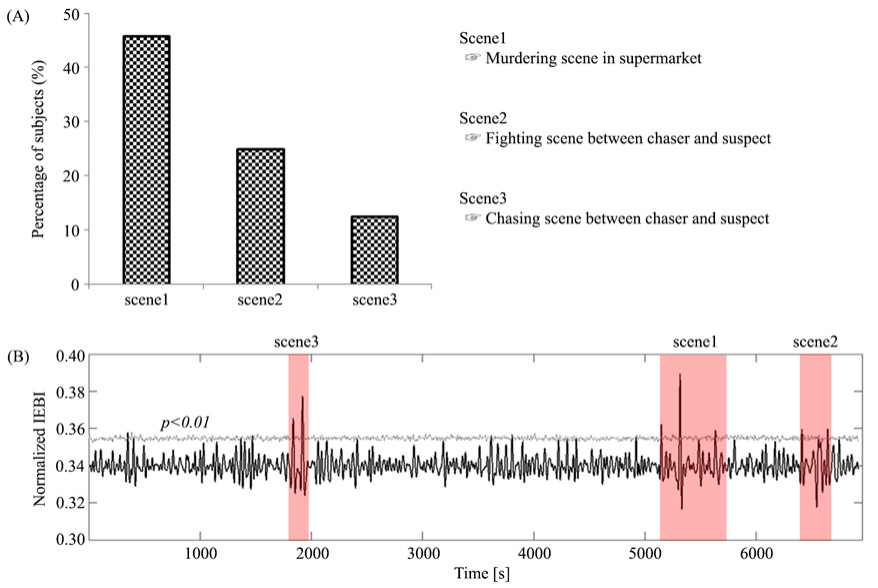
Temporal matching between normalized IEBIs and scenes reported as memorable by subjects. (A) Most memorable scenes: the murder scene was reported as most memorable by 46% of subjects (Fig 3A and S2 Dataset). (B) Changes in the temporal characteristics of normalized IEBIs in all subjects in the MOVIE condition. Red bars indicate the most memorable parts of the movies (Fig 3B and S3 Dataset). The gray line depicts statistical significance (p < 0.01), as determined using a random permutation test.

Immediately after the task, subjects were asked to report the most memorable scene during film watching in order to find the relationship between memorable scenes and the temporal pattern of IEBIs and to understand common interesting scenes across subjects. The murder climax scene was most commonly reported to be the most memorable scene. This scene was selected by 46% percent of all subjects. The second-most memorable scene was a fight sequence between the police and suspect (25%). The third-most memorable scene was the police chase (12%). As shown in Fig 3, the greatest increase in IEBIs coincided with the most memorable scene. In addition, the other significant peaks (p<0.01) matched the second- and third-most memorable scenes in the movie.

### IEBI and episodic memory

**Fig 4:**
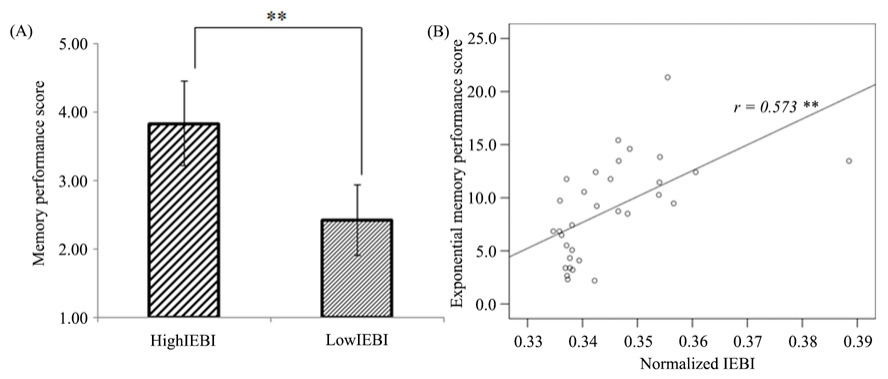
Relationship between IEBI and memory recall after one month. (A) Memory performance for High-IEBI and Low-IEBI scenes. (B) Correlation between normalized IEBI value and memory performance score (**: p< 0.01, Fig 4B and S4 Dataset).

After four weeks, subjects performed the episodic memory test consisting of 32 items. We acquired a memory performance score from Q2 which asked subjects to rate each scene according to how memorable they were using a scale of 1(not at all) to 5 (perfectly). Pearson correlation tests were employed to determine the relationship between IEBI and the memory performance score. As shown in Fig 4A, scenes that coincided with longer IEBIs were more memorable than scenes with lower IEBIs. Accordingly, there was a statistically significant positive correlation between normalized IEBI values and memory performance scores (r = 0.573, N = 32, p<0.001, 2-talied).

## Discussion

This study is the first attempt to elucidate the relationship between IEBI and enhanced episodic memory during movie viewing. We demonstrated that IEBI increased in relation to the memorability of the movie scene. There was a significant correlation between IEBI and enhanced episodic memory during movie watching.

One of the unique analytic features of this study compared to previous eye blink studies is its focus on the timing and duration of suppressed eye blinks. It can be challenging to identify the synchronization of common stimuli with eye blinking and to differentiate spontaneous eye blinks from blinks that occur for other reasons, including the prevention of dry eye, and from reflexive and voluntary blinking. In our study, the average IEBI during movie viewing was significantly greater than that during the nature documentary. The episodic narrative of the movie better captured subjects’ visual attention compared to the documentary clip, which did not have a story line. The results agree with previous research that indicates that mean blink rate is significantly slower while viewing a short video clip with an attractive story than during the rest state [15]. In the MOVIE condition of the present study, the number of long IEBIs (> 8 sec) also increased significantly, with a few subjects not blinking for more than one minute, as depicted in Fig 2. While watching interesting scenes in a movie, eye blinks can be spontaneously suppressed for long periods of time in order to increase visual attention. There is a known association between spontaneous eye blinking and visual attention [23–25]. This relationship between interesting or memorable moments in movies and eye blink suppression was also confirmed by analyzing the temporal pattern of IEBIs and by asking subjects to score their recall of particular scenes immediately after watching the movie. The scene reported as most memorable by subjects coincided exactly with the most significant peak in the average IEBI of all subjects, as shown in Fig 3. In addition, the other significant IEBI peaks corresponded to the scenes in the movie scored as second- and third-most memorable. Because the IEBIs of all subjects were averaged, the significant change in the timing of the peak IEBI indicated that IEBI was highly consistent across subjects at common interesting parts of the movie. Within brain networks, eye blinking is suppressed by the superior colliculus (SC) [26, 27], which is under the control of higher cortical areas such as the supplemental eye field, frontal eye field, and posterior parietal cortex (PPC). The PPC is primarily related to eye blinking [28, 29] and attentional processing [30], especially in the process of disengaging covert visual attention [31]. These previous findings also indicate that excellent control of blink suppression may be closely related to the visual attention system, which contributes to stable visual perception and awareness through the interruption of eye blinking.

On the other hand, we could consider that eye blink could be associated with rate of presentation of new visual information. Some researchers have found this relationship between eye blink and fast changing visual information [32, 33]. Cardona and Quevedo used VDT tasks that required subjects to play two different computer games: one fast-paced game and another slow-paced game. The results indicated that blink rate during fast-and slow-paced computer game play decreased to almost 1/3 and 1/2 of baseline levels, respectively. That is, if information changes very fast subjects do not blink to avoid losing important information during the 0.4 s blackout time associated with a blink. Because the scenes reported as most memorable included a murder, a fight and a police chase, the association of eye blink and episodic memory could be interpreted in that more visually rich, dynamic or challenging scenes are better remembered because blinking is avoided during that time.

The close relationship between IEBI and visual attention could be extended to an association with successful memory, considering that the formation of enduring memory depends on attention [34, 35]. Our results demonstrated that there was a significant positive correlation between IEBI and subsequent memory encoding (Fig 4B). That is, scenes that coincided with long IEBIs were better remembered than those that coincided with short IEBI. It has been well established that attention improves memory. A few studies have also suggested that visual attention is important for maintaining information in short-term memory [36–38]. Irwin has reported that eye blinks reduce short-term memory capacity for feature and conjunction stimuli because visual information was physically blocked [39]. In our study, when the subjects were asked to describe the most highly scored scene, they recalled the events of the story that took place before and after the scene. Therefore, we believe our results show that the modulation of visual attention by eye blink suppression affects successful episodic memory encoding.

There were a few limitations in our experimental procedure. First, we did not fully consider dry eye in terms of evaluation or diagnosis. Although we excluded subjects using contact lenses and kept the humidity at approximately 50% to prevent fatigue or dryness by contact lenses, dry eye may influence to spontaneous blinks because a representative function of spontaneous eye blink is to keep the eyeball lubricated and cleansed. Second, fatigue may have an effect on the intrinsic differences between watching a 2h movie and a 5 minute documentary. It has been documented that sleepiness leads to changes in eye blink rate and IEBI [40]. First signs of sleepiness were reported as sporadic periods of long eyelid closure. In this study, video monitoring was used to check sleepiness during the experiment and no one fell asleep. In light of these facts, fatigue had little effect on our experimental results. Nevertheless, in further research, we need to consider the objective assessment method for evaluating the effect of visual fatigue on the eye blink. Finally, we could consider the effect of sound level. In this study, the acoustic pressure level was measured during movie and documentary watching. Although there was no significant difference between MOVIE (31.1 ± 12.2dB) and CONTROL (28.3 ± 12.1dB), we could not rule out the possibility that strong sound may lead to reflex blinking resulting in changes in eye blink rate between MOVIE and CONTROL conditions.

In conclusion, we demonstrated that significantly longer IEBIs during movie watching are correlated with the most memorable scenes. In addition, the subjects remembered the scenes associated with long IEBIs better than scenes associated with short IEBIs, indicating that IEBI could be used as a reliable bio-marker of subjects’ concentration on content that requires visual attention, such as a movie.

## Supporting Information

S1 Dataset Mean IEBI of each subject in conditions. (Fig 2A)
(XLSX)

S2 Dataset The result of survey for the most memorable scene. (Fig 3A)
(XLSX)

S3 Dataset Time series data of normalized IEBI (filtered by Gaussian kernel) from raw of IEBI time series data in movie condition. (Fig 3B)
(XLSX)

S4 Dataset Memory performance score and normalized IEBIs at the scene. (Fig 4B)
(XLSX)
